# PhenoFit: a framework for determining computable phenotyping algorithm fitness for purpose and reuse

**DOI:** 10.1093/jamia/ocaf195

**Published:** 2025-11-12

**Authors:** Laura K Wiley, Luke V Rasmussen, Rebecca T Levinson, Jennnifer Malinowski, Sheila M Manemann, Melissa P Wilson, Martin Chapman, Jennifer A Pacheco, Theresa L Walunas, Justin B Starren, Suzette J Bielinski, Rachel L Richesson

**Affiliations:** Department of Neurology, Institute for Informatics, Data Science, and Biostatics, Washington University in St. Louis School of Medicine, St. Louis, MO 63110, United States; Department of Preventive Medicine, Northwestern University Feinberg School of Medicine, Chicago, IL 60611, United States; Department for General Internal Medicine and Psychosomatics, Heidelberg University Hospital, Heidelberg, 69120, Germany; Write InScite LLC, Pound Ridge, NY 10576, United States; Division of Epidemiology, Department of Quantitative Health Sciences, Mayo Clinic, Rochester, MN 55905, United States; Division of Infectious Diseases, Department of Medicine, University of Colorado Anschutz Medical Campus, Aurora, CO 80045, United States; Department of Population Health Sciences, King’s College London, London, WC2R 2LS, United Kingdom; Center for Genetic Medicine, Northwestern University Feinberg School of Medicine, Chicago, IL 60611, United States; Division of General Internal Medicine and Center for Health Information Partnerships, Department of Medicine, Institute for Public Health and Medicine, Northwestern University Feinberg School of Medicine, Chicago, IL 60611, United States; Center for Biomedical Informatics and Biostatistics, University of Arizona, Tucson, AZ 85721, United States; Division of Epidemiology, Department of Quantitative Health Sciences, Mayo Clinic, Rochester, MN 55905, United States; Department of Learning Health Sciences, University of Michigan Medical School, Ann Arbor, MI 48109, United States

**Keywords:** computational phenotyping, cohort identification, EHR, fitness for purpose

## Abstract

**Background:**

Computational phenotyping from electronic health records (EHRs) is essential for clinical research, decision support, and quality/population health assessment, but the proliferation of algorithms for the same conditions makes it difficult to identify which algorithm is most appropriate for reuse.

**Objective:**

To develop a framework for assessing phenotyping algorithm fitness for purpose and reuse.

**Fitness for Purpose:**

Phenotyping algorithms are fit for purpose when they identify the intended population with performance characteristics appropriate for the intended application.

**Fitness for Reuse:**

Phenotyping algorithms are fit for reuse when the algorithm is implementable and generalizable—that is, it identifies the same intended population with similar performance characteristics when applied to a new setting.

**Conclusions:**

The PhenoFit framework provides a structured approach to evaluate and adapt phenotyping algorithms for new contexts increasing efficiency and consistency of identifying patient populations from EHRs.

## Introduction

Identifying patient cohorts from electronic health records (EHRs) is critical for research, clinical decision support, and quality/population health assessment. This process, called computational phenotyping, uses multiple types of EHR data to infer the phenotype (eg, clinical condition or traits) of a patient and can employ any combination of rule-based logic, natural language processing (NLP), and/or machine learning methods.^1^ The breadth of application domains has led to each community developing their own processes and best practices for phenotyping algorithms.^2^ This has caused a proliferation of algorithms for the same phenotypes, with systematic literature reviews identifying 66 algorithms for asthma,^3^^,^^4^ 30 for heart failure,^5^ and 17 for acute myocardial infarction.^6^ Importantly, these algorithms do not always identify clinically equivalent populations despite nominally being developed for the same phenotype,^7–10^ potentially introducing heterogeneity into scientific and clinical practice. Increasing phenotype algorithm reuse would not only reduce algorithm development cost, but also improve research efficiency and reproducibility.

Computational phenotype libraries that centralize storage of definitions and code, offer one potential solution to encourage phenotype algorithm reuse. Several research communities—including the eMERGE Phenotype Knowledge Base (PheKB),^11^ the OHDSI Phenotype Library,^12^ the MVP Phenotype Library,^13^ Centralized Interactive Phenomics Resource (CIPHER),^14^ and others^15^^,^^16^—have developed phenotype libraries and metadata elements^17^^,^^18^ to support their networks. However our evaluation found existing metadata to be inconsistent and incomplete.^19^ Further, while these libraries are accessible, they are often not easily findable meaning potential users would need to have both insight and time to manually search multiple network and collaboration sites for phenotype definitions. Even within individual libraries, multiple definitions for the same phenotype exist, emphasizing the need for descriptive metadata to help researchers choose the most appropriate algorithm for their purpose.

Although there is a need for algorithm standardization and reuse, in practice multiple algorithms will likely always be needed to capture the full spectrum of phenotype presentations and deployment contexts. Accordingly there will always be a need for researchers to evaluate algorithm quality and appropriateness for reuse.^2^^,^^20^^,^^21^ In this work, we define a framework for establishing whether a phenotyping algorithm is “fit for purpose” and provide guidance to investigators on how to use this framework to assess and compare existing algorithms to determine which is most appropriate for reuse in their specific target context.

## Component 1: assess fitness for purpose

To determine whether a phenotyping algorithm is fit for purpose, it is critical to understand the specific population it targets and how reliably it detects that target population.

### Intended population

The complexity of phenotype development context is often obfuscated by our labeling algorithms only by disease/condition name. While these names are rarely inaccurate, they may not fully communicate the full nuance of the intended phenotype definition targeted by the algorithm. For example, our recent systematic review found more than 23 different phenotyping algorithms for “heart failure,”^5^ yet there are many different definitions of heart failure used in clinical practice guidelines,^22–24^ epidemiological research,^25^^,^^26^ and clinical trials.^27–35^ There are also many subclassification approaches including separating patients based on their left ventricular ejection fraction or symptom severity/exacerbation. While these definitions share many similarities, patients may be identified as having heart failure by one set of criteria but not another.^36^ While some algorithms explicitly identify which of these definitions they are targeting many do not, and it stands to reason that phenotyping algorithms will have differential performance in identifying patients who meet any particular disease clinical definition. This has been shown in acute myocardial infarction where algorithms had 94.6% positive predictive value (PPV) for identifying patients who met AHA Council of Epidemiology and Prevention criteria but only 65.3% PPV for patients who met WHO MONICA criteria.^10^ Additionally, algorithms may be intended to detect a broad phenotype (ie, “diabetes”) but may preferentially detect a specific subtype (eg, type 2 diabetes) or explicitly exclude certain subtypes (eg, gestational diabetes).^7^

When selecting an existing phenotyping algorithm for reuse, investigators should carefully review the specific population targeted and identified during the initial development. The most accurate definition of the intended population is the definition used to adjudicate “true positive” patients during validation. However, in practice this is rarely provided. In the case of heart failure, only 5 of the 23 algorithms provided information about their validation strategy with 3 using some combination of the Framingham criteria to define “true” heart failure patients.^5^ In some cases, developers may explicitly state the specific phenotype under development. For example, the OHDSI Phenotype library recommends including a description of the clinical or biological definition of the phenotype targeted by the algorithm.^37^ While this is helpful to understand intent, without information on how that definition was operationalized during validation there is no way to guarantee it is an accurate reflection of the patient population identified. There are also new approaches using empirical examination of population characteristics to aid in evaluating the algorithm definition,^38^ though this approach may be highly influenced by the source population used for algorithm development. When no details are provided about the target population investigators may assess the face validity of the algorithm components for their desired population recognizing that there are very strong limitations to this approach.

Finally, investigators should exercise caution to ensure there are not “hidden influencers” in the algorithm design. For example, numerous “heart failure” algorithms included a filter requiring an ejection fraction less than 40%. While this does identify a heart failure population as stated in the original publication, in practice it will only ever detect patients with the reduced-ejection fraction subtype. These “hidden influencers” in algorithm design may cause a mismatch between the captured population and what an investigator might expect based on the algorithm name or stated target population. Finally, based on the original development context, some algorithms may also include “control” or “non-case” definitions with their algorithm. There are many different types of controls (clinical, biological, etc.) and control algorithms are highly dependent on deployment context. While evaluation of these definitions is critical, they are outside the scope of the PhenoFit framework which is exclusively designed to evaluate case definitions.

### Appropriate performance

Rigorously developed algorithms are validated and report the performance of the algorithm using statistics such as positive/negative predictive value (PPV/NPV), sensitivity, specificity, AUC, and F1 measure. Ideally algorithms would have perfect performance across all of these metrics, however in practice developers usually have to select only 1 or 2 measures on which to optimize performance. Common scenarios include selecting for high PPV (ie, high proportion of algorithm-identified cases are true cases), high specificity (ie, correctly identifying people who do not have the phenotype, minimizing false positives), or high sensitivity (ie, correctly identifying all people with the phenotype, minimizing false negatives). The optimal performance measure is highly dependent on the intended purpose of algorithm development. For example, in clinical trial recruitment, if you plan to send direct recruitment mailings to all patients identified by the phenotyping algorithm you would want to optimize for high PPV (so that you do not accidentally send materials to someone without the phenotype of interest).^39^ In contrast, if you are studying a rare disease, you likely will want to optimize for sensitivity to ensure you capture all possible individuals. Although this will likely come at the cost of increased numbers of false-positive individuals identified, the total numbers of individuals identified will likely be small enough to allow for final chart review adjudication.

Prior to selecting an algorithm for reuse, investigators should think carefully about their application context, identifying which performance measure(s) are most important for their goals. Ideally the algorithm they select should have high performance for the selected measure, though additional details about the original evaluation context should be considered. First, PPV is highly sensitive to population prevalence, so algorithms developed or validated in an enriched population will have inflated performance than when it is applied to a general population. Similarly, sensitivity and specificity performance can be inflated when uncertain/difficult to adjudicate cases are removed from the gold standard, an example of spectrum bias. Finally, many algorithms have either never been validated or the performance characteristics not reported. In these cases it is impossible to assess the algorithm’s fitness for purpose beyond a rough assessment of face validity when specific components of the algorithm are described. While this does not preclude reuse of these algorithms, investigators should exercise caution as the algorithm may not be suitable for their application context.

## Component 2: assess fitness for reuse

**Figure 1.**
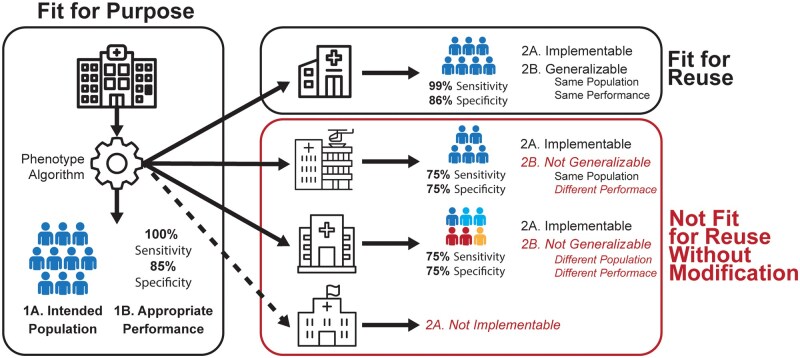
PhenoFit framework applied to 4 fitness for reuse scenarios. First, a computational phenotyping algorithm must be fit for purpose (Component 1) that is it identifies the intended population (Component 1A) with appropriate performance (Component 1B). In this case, the algorithm needs to identify blue people with high sensitivity. To be fit for reuse (Component 2), the algorithm must be implementable (Component 2A) and generalizable (Component 2B). The first scenario (top) is fit for reuse because it was implementable and identified blue people with high sensitivity. In the second scenario the algorithm was implementable but was not generalizable because although it identified blue people it no longer had high sensitivity. The third scenario similarly lacks generalizability because it identified more than just blue people and did not have high sensitivity. Finally in the fourth scenario (bottom) the algorithm was not implementable. Scenarios 2-4 are not fit for reuse unless the algorithm is modified to become fit for purpose at the new site.

**Figure 2.**
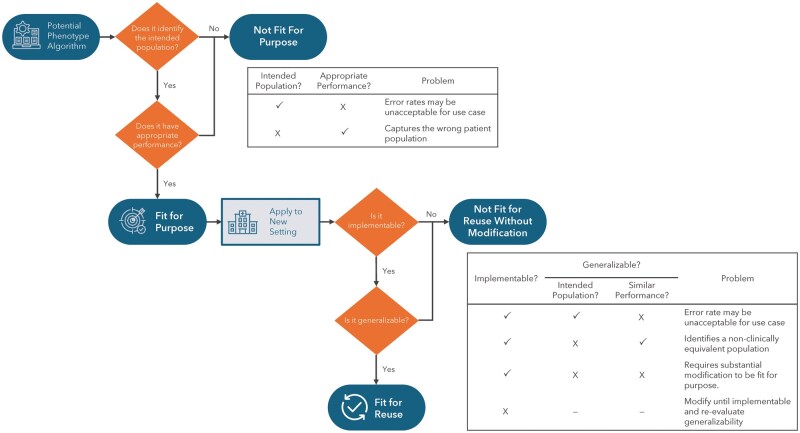
PhenoFit evaluation flowchart. Application of the PhenoFit framework to a new algorithm to determine fitness for purpose and/or reuse. Where an algorithm is determined to not be fit for purpose and/or reuse a decision table describes the type of potential problem.

Once an algorithm has been determined to be fit for purpose, it must also be implementable and generalizable to be fit for reuse. Figure 1 shows the PhenoFit framework applied to 4 different reuse scenarios. Although the algorithm is considered fit for reuse in only a single scenario (where it is fit for purpose, implementable, and generalizable) any algorithm may be used as an initial starting point and then modified as necessary to achieve implementability and/or generalizability. This form of reuse with modification often has the benefit of reduced implementation time/effort compared to complete de-novo development. A flow chart of these steps is shown in Figure 2.

### Implementable

While it seems a given that an existing algorithm will be clearly defined, in reality phenotyping algorithms have widespread underspecification that leads to ambiguity and variation that can substantially affect the implementability of an algorithm at a new site.^40^ For example, a diabetes phenotyping algorithm using “any abnormal hemoglobin A1c” as a criteria would require investigators to make their own quantification of the threshold for “abnormal” which could potentially differ from the original implementation. Machine learning or statistical models require model weights, intercepts, and variable definitions be available from the original algorithm development. Likewise, NLP algorithms need details on the original keywords or regular expressions, and may or may not perform well given local documentation practices.^41^ Investigators should carefully assess the availability of the source algorithm and pay close attention to potential areas of ambiguity that will need to be resolved during the implementation process. In some cases a phenotype algorithm may simply be unimplementable at the reuse site.

Another critical factor to algorithm implementability is whether the data and technology/skillset are available at the site of reuse. Every EHR has slight differences in how/where data are stored and whether those data are easily available for reuse. For example, although all EHRs have the ability to capture clinical notes, not all data warehouses make clinical notes available to researchers due to patient privacy protections. In such a case an NLP-based algorithm would not be suitable for reuse since notes are not accessible. In other cases data may be available but not coded with the same terminology used in the source algorithm. For example the algorithm may use LOINC identifiers for laboratory tests but investigators only have access to lab names. While this is often surmountable, it dramatically increases implementation time and required expertise. Expertise and technology gaps also affect implementability. For example, some algorithms may be distributed as R or Python code. Investigators may be limited to performing SQL queries of their data warehouse or may not have team members with experience in R or Python to be able to directly implement the source phenotyping algorithm. Common data models, among other solutions,^42–45^ offer a valuable approach to address implementation barriers by providing a common data structure/vocabulary and shared executable code for each algorithm. However, in practice, many of these challenges still exist due to sites not fully/compliantly implementing the CDM and many historical phenotyping algorithms do not yet have a CDM-based implementations. Investigators should carefully consider what data, technology, and expertise they have available when selecting an algorithm for reuse to ensure implementability.

### Generalizable

In epidemiology, generalizability refers to the extent to which the results of an investigation can be applied to broader populations or contexts beyond the specific conditions of the original study. In phenotyping we propose that generalizability refers to whether phenotyping algorithms retain their performance when applied in new contexts. That is they remain fit for purpose, identifying the same intended population with the same optimization characteristics as in their development site. Most research around phenotype reuse to date has focused solely on portability/implementability and so there is limited data around the conditions/characteristics that affect algorithm generalizability. As such, there are currently no methods to *a priori* identify whether an algorithm will be generalizable. Therefore, investigators should always be prepared to perform at least a minimal validation of the algorithm at their site to confirm it is fit for their purpose. For algorithms that have not previously been validated (or have no reported performance characteristics) we recommend more robust validation studies. If the algorithm does not perform as expected then it should be modified and revalidated until it achieves the desired performance.

There are many reasons why an algorithm may not be generalizable. As described above, original validation approaches/contexts may inflate apparent statistical performance. There may also be key structural differences between the source and reuse populations that will affect the types of patients seen or care provided. For example, a phenotyping algorithm for breast cancer developed at a comprehensive cancer center with surgery, radiology, and chemotherapy services is unlikely to be generalizable to a regional hospital that does not offer such specialized cancer services as phenotyping algorithms can be highly sensitive to these types of healthcare process differences.^46^^,^^47^ An additional consideration affecting generalizability is the types of filters applied to the data source prior to phenotype algorithm development. For example, many phenotyping algorithms developed by the eMERGE consortium^48^ are filtered to only those patients who have enrolled in institutional biobanks. While the algorithms are performant in that selected set of individuals, they may not retain performance when applied to the general patient population. When selecting algorithms for reuse, investigators should pay close attention to the original development context and any filters applied to the source population. The more different these features are between development and reuse populations, the less likely the algorithms are to generalize without requiring extensive modification. This alignment between source development and reuse context becomes particularly important when investigators are unable to validate algorithm performance in their dataset. In this situation investigators should recognize that they may have unknown levels of phenotype misclassification and adapt their usage accordingly.

## Additional considerations

We have proposed a framework for evaluating fitness for purpose and fitness for reuse of phenotype algorithms. Although this framework provides a useful conceptual model there are additional considerations that affect the practicalities of algorithm reuse.

1. *Temporality—*fitness for use and reuse are likely to vary over time. For example, clinical definitions of disease are under constant change as clinical guidelines are updated, and the “intended population” for a study today may no longer comport with our understanding of the same disease 5 years from now. Similarly, technologies, terminologies, and documentation practices are dynamic and constantly evolving affecting both the implementability and generalizability of algorithms over time.^49^
2. *Multi-site usage—*Consortia often select a single algorithm to apply across all sites, but due to differences in implementability or generalizability these algorithms may not perform consistently across sites. Although a core algorithm may be used as a shared starting point, we recommend all sites first align on fitness for use goals (ie, intended population and appropriate performance). This allows for necessary site specific customizations to ensure each site’s algorithm is fit for purpose. We also note that some consortia a priori have different performance goals due to differences in study design.^39^ In these situations, algorithms are considered equivalently fit for purpose if they all identify the same intended population.
3. *Data only phenotyping—*It is increasingly common that phenotype algorithms are deployed in contexts where traditional validation methods are not possible (such as large deidentified data networks). Fundamentally the accuracy of algorithms that use data with uncertain fidelity share that uncertainty. While there is a cost-benefit calculation between the risk of phenotype misclassification vs. the benefit of these unique data resources, users need to be aware of and report this uncertainty. Although PhenoFit cannot assess fitness in these contexts, it provides a useful construct for contextualizing the types of uncertainty present when evaluating the quality of data only algorithms. For example, phenotype algorithms that have already been validated and reused across multiple contexts with high fidelity are likely to be similarly high performing in a data-only environment. Tools like CohortDiagnostics^38^ provide a lens for evaluation of the intended population, while sensitivity analyses can provide assurance around the boundaries of appropriate performance.
4. *Reporting guidelines and metadata standards are needed—*Although there are hundreds of phenotyping algorithms published in the literature, many do not provide sufficient details about the algorithm to allow for reuse. Similarly, there are no agreed upon metadata standards for phenotype libraries, so even when the algorithms are made available they may lack sufficient details about implementation context to fully evaluate fitness for purpose/reuse.

## Data Availability

No new data were generated or analyzed in support of this research.
